# Reversible Tau Phosphorylation Induced by Synthetic Torpor in the Spinal Cord of the Rat

**DOI:** 10.3389/fnana.2021.592288

**Published:** 2021-02-02

**Authors:** Timna Hitrec, Fabio Squarcio, Matteo Cerri, Davide Martelli, Alessandra Occhinegro, Emiliana Piscitiello, Domenico Tupone, Roberto Amici, Marco Luppi

**Affiliations:** ^1^Department of Biomedical and NeuroMotor Sciences, University of Bologna, Bologna, Italy; ^2^Department of Neurological Surgery, Oregon Health & Science University, Portland, OR, United States

**Keywords:** hypothermia, hibernation, microglia, tauopathies, GSK3β, motor neurons, adaptive response

## Abstract

Tau is a key protein in neurons, where it affects the dynamics of the microtubule system. The hyperphosphorylation of Tau (PP-Tau) commonly leads to the formation of neurofibrillary tangles, as it occurs in tauopathies, a group of neurodegenerative diseases, including Alzheimer's. Hypothermia-related accumulation of PP-Tau has been described in hibernators and during synthetic torpor (ST), a torpor-like condition that has been induced in rats, a non-hibernating species. Remarkably, in ST PP-Tau is reversible and Tau de-phosphorylates within a few hours following the torpor bout, apparently not evolving into pathology. These observations have been limited to the brain, but in animal models of tauopathies, PP-Tau accumulation also appears to occur in the spinal cord (SpCo). The aim of the present work was to assess whether ST leads to PP-Tau accumulation in the SpCo and whether this process is reversible. Immunofluorescence (IF) for AT8 (to assess PP-Tau) and Tau-1 (non-phosphorylated Tau) was carried out on SpCo coronal sections. AT8-IF was clearly expressed in the dorsal horns (DH) during ST, while in the ventral horns (VH) no staining was observed. The AT8-IF completely disappeared after 6 h from the return to euthermia. Tau-1-IF disappeared in both DH and VH during ST, returning to normal levels during recovery. To shed light on the cellular process underlying the PP-Tau pattern observed, the inhibited form of the glycogen-synthase kinase 3β (the main kinase acting on Tau) was assessed using IF: VH (i.e., in motor neurons) were highly stained mainly during ST, while in DH there was no staining. Since tauopathies are also related to neuroinflammation, microglia activation was also assessed through morphometric analyses, but no ST-induced microglia activation was found in the SpCo. Taken together, the present results show that, in the DH of SpCo, ST induces a reversible accumulation of PP-Tau. Since during ST there is no motor activity, the lack of AT8-IF in VH may result from an activity-related process at a cellular level. Thus, ST demonstrates a newly-described physiological mechanism that is able to resolve the accumulation of PP-Tau and apparently avoid the neurodegenerative outcome.

## Introduction

In neurons, Tau is a key protein involved in the functional regulation of the microtubule system and belongs to the wider family of microtubule-associated proteins (Wang and Mandelkow, 2016). The functional role of Tau is mainly modulated by phosphorylation/dephosphorylation processes, which are finely regulated and targeted toward different sites of the amino-acidic sequence (Wang and Mandelkow, 2016). In recent years, Tau has represented a focus of interest since it is primarily involved in many neurodegenerative diseases (among them, Alzheimer's disease), commonly defined as tauopathies (Gerson et al., 2016; Kovacs, 2017). Apparently, in these cases the pathologic neuronal death is triggered by the accumulation of a hyper-phosphorylated form of Tau (PP-Tau), that detaches from microtubules and tends to aggregate in oligomers firstly and then into neurofibrillary tangles (Gerson et al., 2016).

The accumulation of PP-Tau has also been described in non-neurodegenerative conditions in either hibernating mammals (Arendt et al., 2003, 2015) or mice exposed to physiological challenges (Planel et al., 2001, 2007; Okawa et al., 2003). In all these cases, the hyper-phosphorylation was reversible and did not apparently evolve toward neurodegeneration. Recently, a reversible accumulation of PP-Tau has been shown in the rat during “synthetic torpor” (ST; Cerri et al., 2013; Cerri, 2017), a torpor-like state which is induced in a non-hibernator by means of the central nervous pharmacological blockade of thermogenesis.

In all these “PP-Tau reversible” conditions, to the best of our knowledge no data have been shown regarding divisions of the nervous system other than the brain. In tauopathies, the involvement of PP-Tau in the spinal cord (SpCo) is of interest. Guo et al. (2016) showed that, in humans, the SpCo appears to be interested by neurofibrillary tangle accumulation. Moreover, in transgenic mice models expressing human Tau mutations that evolve into tauopathies, i.e., P301L (Lewis et al., 2000) and P301S (Allen et al., 2002), SpCo is dramatically affected.

Since in Luppi et al. (2019) the study focused on the brain, a dedicated study on the SpCo appears of particular interest concerning two main objectives: (i) to integrate and complete the knowledge of phosphorylation/dephosphorylation processes of Tau protein in the central nervous system during ST; (ii) to better define the possible parallelism of these processes with those occurring in tauopathies. Therefore, we sought to assess the expression of PP-Tau in the SpCo of rats, during both the induction of ST and the following recovery period.

Moreover, since tauopathies appear to be closely related to neuroinflammation (Ransohoff, 2016; Nilson et al., 2017), as a preliminary investigation of this process in the SpCo we also assessed the microglia activation during the experiment.

## Materials and Methods

### Animals

The animals used for the present work were the same as those used in the experiment described in Luppi et al. (2019). In contrast, however, the present work is focused on the SpCo, a division of the nervous system that was not considered in our previous analysis.

A total of 17 Male Sprague–Dawley rats (201–225 g; Charles River) were used. Animals were acclimated to normal laboratory conditions: ambient temperature (Ta) set at 24±0.5°C; 12 h:12 h light-dark (LD) cycle (L: 09:00 h−21:00 h; 100–150 lux at cage level); food and water *ad libitum*. All the experiments were conducted following the approval by the National Health Authority (decree: No. 112/2018-PR), in accordance with the DL 26/2014 and the European Union Directive 2010/63/EU, and under the supervision of the Central Veterinary Service of the University of Bologna. All efforts were made to minimize the number of animals used and their pain and distress.

### Surgery

The procedure has been previously described (Cerri et al., 2013). Briefly, deeply anesthetized rats (Diazepam, 5 mg/kg i.m.; Ketamine-HCl, 100 mg/kg i.p.) placed in a stereotaxic apparatus (David Kopf Instruments) were surgically implanted with the following: (i) electrodes for electroencephalogram (EEG) registration; (ii) a thermistor (Thermometrics Corporation) stereotaxically placed beside the left anterior hypothalamus to record deep brain temperature (Tb); (iii) a microinjection guide cannula, targeted to the Raphe pallidum, at the following stereotaxic coordinates from lambda: on the midline, 3.0 mm posterior and 9.0 ventral to the dorsal surface of the cerebellum (Paxinos and Watson, 2007). After surgery, animals received subcutaneous saline and intramuscular antibiotic solution. Each rat recovered from surgery for at least 1 week. Prior to the experimental session rats were placed in a cage positioned within a thermoregulated and sound-attenuated chamber. During this 3-day adaptation period, rats were exposed to a mild low Ta (15°C), constant darkness and were fed a high-fat diet (35% fats, Mucedola), conditions that are known to favor the occurrence of a torpid state in hibernators (Cerri et al., 2013).

### Synthetic Torpor

To induce ST, we used a consolidated protocol (Cerri et al., 2013; Luppi et al., 2019; Tinganelli et al., 2019). Briefly, a microinjecting cannula was inserted into the implanted guide cannula. Then, 100 nl of 1 mM muscimol (a GABA_A_ agonist) was injected once an hour, six consecutive times. Following the last injection, Tb reached values of around 22°C (Cerri et al., 2013). One hour after the last injection, Ta was set at 28 ± 0.5°C to favor the return to normothermia of the animal. As a control group, three animals were injected with artificial cerebrospinal fluid (aCSF; EcoCyte Bioscience). During the whole experiment, EEG and Tb signals were recorded, after being opportunely amplified, filtered, and digitalized (Cerri et al., 2013), with the aim of better monitoring animals' behavior during ST induction and in the following recovery period.

### Experimental Procedure

**Figure 1:**
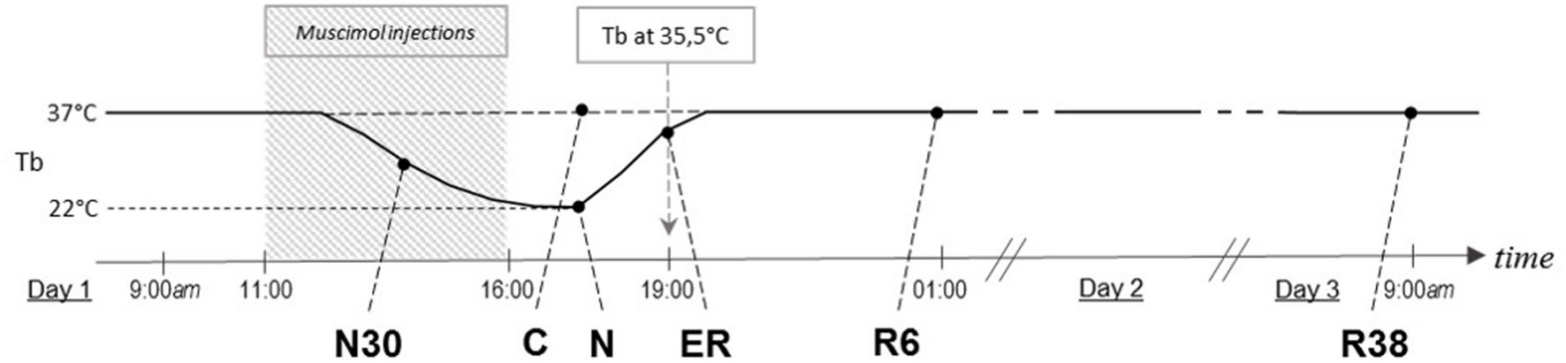
Schematic representation of how the experimental conditions were defined. The solid line indicates the progress of brain temperature (Tb) throughout the experiment. The dotted line refers to the control group (C). The shaded area represents the period when synthetic torpor (ST) was induced. N30, samples taken when Tb reached 30°C; N, samples taken at nadir of hypothermia, during ST; ER, early recovery, samples taken when Tb reached 35.5°C following ST; R6, samples taken 6 h after ER; R38, samples taken 38 h after ER.

Animals were randomly assigned to six different experimental groups and were sacrificed at different times following the injection of either muscimol or aCSF (first injection at 11.00 h). A representative scheme of the protocol is shown in Figure 1. The experimental groups were the following:

- **C** ➔ Control, injected with aCSF (*N* = 3) and sacrificed at around 17.00 h, exactly matching the N condition.
- **N30** ➔ sacrificed at around 12.00 h−13.00 h, between the second and third injection of muscimol, when Tb reached the level of 30°C (*N* = 3).
- **N** ➔ sacrificed 1 h after the last injection, at 17.00 h, when Tb reached the nadir of hypothermia (*N* = 3; Tb = 22.1 ± 1.4°C).
- **ER** ➔ early-recovery; sacrificed at around 19.00 h (2 h after Ta was moved from 15 to 28°C) when Tb reached 35.5°C after ST; at this specific point of the protocol, animals began to show clear signs of sleep at the EEG level (*N* = 3).
- **R6** ➔ sacrificed at around 01.00 h, 6 h after ER (*N* = 3).
- **R38** ➔ sacrificed at around 09.00 h of the third day, 38 h after ER (*N* = 2).

### Immunofluorescence/Histology

At the different time points of the experimental protocol, rats under general anesthesia were transcardially perfused with 200 ml of saline solution (NaCl 0.9%, w/v) followed by an equal amount of 4% (w/v) paraformaldehyde solution in sodium phosphate buffer (PBS), both solutions were at room-temperature. The SpCo were extracted and post-fixed for 2 h by immersion in the same solution used for the perfusion, at 4°C. Then, the tissue was put overnight at 4°C in a 30% (w/v) sucrose solution in PBS and sodium-azide 0.02% (w/v) for cryoprotection. Hereafter, tissue samples were embedded in a cryostat cutting medium (Killik) and cut into 35 μm-thick coronal slices, using a cryostat-microtome (Frigocut 2800) set at −22.0°C.

**Figure 2:**
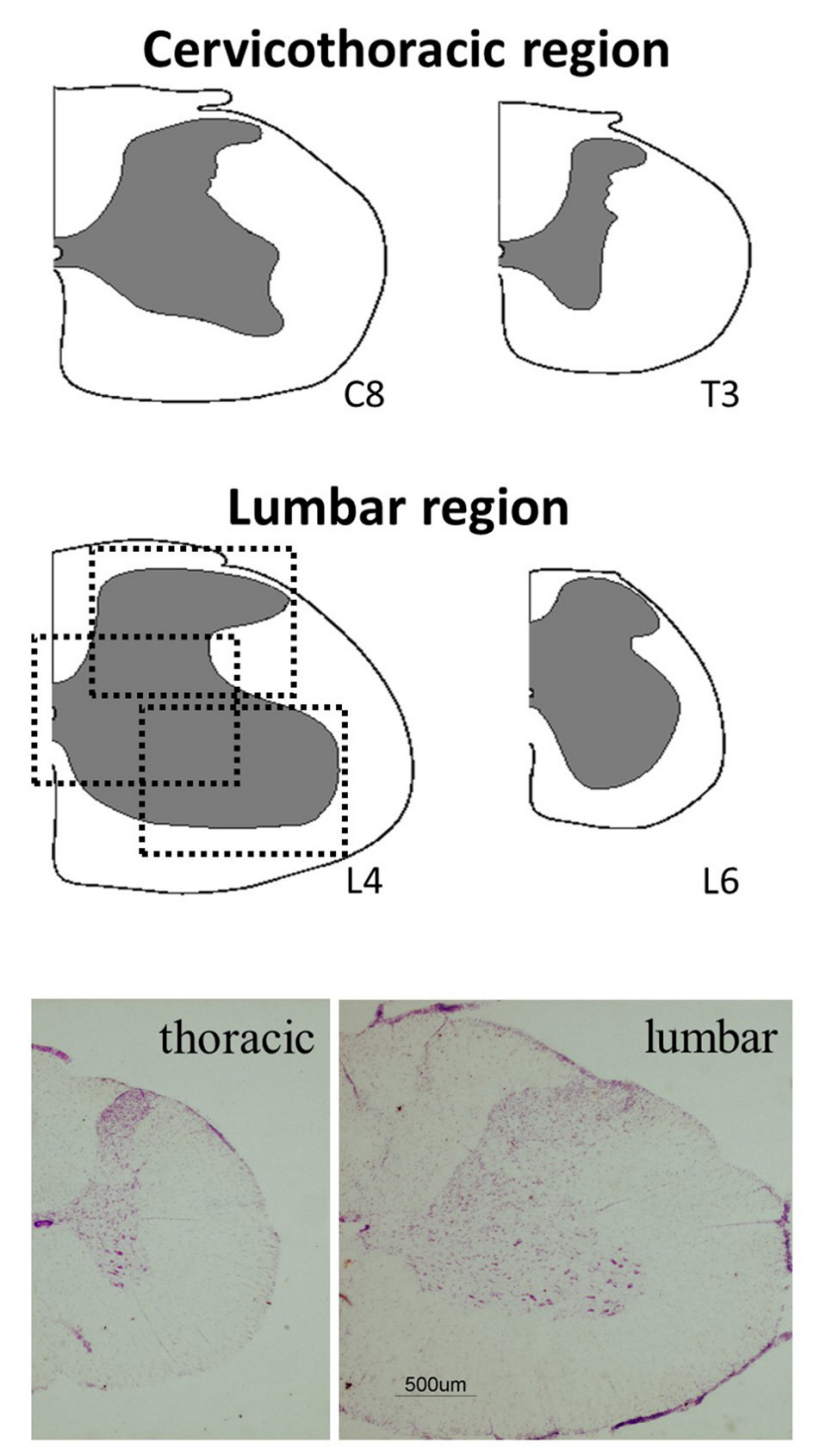
Anatomical areas of interest considered for the analysis. Upper and middle panels show schematic representations of the different levels of the spinal cord considered, and dotted squares show an example of the analyzed fields for each coronal section: dorsal horn, central canal, and ventral horn. Lower panels show low-magnification half coronal sections at representative levels of the spinal cord stained with cresyl violet. Calibration bar: 500 μm.

To sample different SpCo levels, for each immunoreaction section and for each animal we collected 10 slices from the cervicothoracic region and another 10 slices from the lumbar region (Figure 2). In order to better define the anatomical levels, a subset of 50 μm-thick coronal slices from both levels were stained with cresyl violet (Sigma) as described for a previous experiment (Dentico et al., 2009) (Figure 2).

Slices were rinsed twice in PBS and then incubated for 2 h with the blocking solution [1% (v/v) normal donkey serum]. Consequently, all slices were incubated overnight with the following primary antibodies: (i) monoclonal rabbit Anti-NeuN (Merck-Millipore), a neuronal marker; (ii) monoclonal mouse AT8 (Thermo Fisher), marker of the phospho-[Ser202/Thr205]-Tau protein. Both primary antibodies were diluted at 1:400. Slices were then rinsed twice in PBS with 0.3% (v/v) Triton X-100 and incubated with the following secondary antibodies: (i) Donkey Anti-rabbit IgG conjugated with Alexa-488 (Thermo Fisher); (ii) Donkey Anti-mouse IgG conjugated with Alexa-594 (Thermo Fisher). Both secondary antibodies were diluted at 1:500. With the aim to reduce fluorescence fading, SpCo samples were mounted on glass slices using an anti-fade mounting medium (ProLong Gold mountant; Thermo Fisher). All procedures were carried out at room-temperature.

As a control for the AT8 detection, the same procedure was carried out using the monoclonal mouse Tau-1 (Merck-Millipore; 1:400), followed by a Donkey Anti-mouse IgG conjugated with Alexa-594 (1:500). This primary antibody detects Tau protein when it has no phosphorylation between residues from 189 to 207 (Szendrei et al., 1993; Billingsley and Kincaid, 1997).

A sample of sections from C, N30, N, and R6 was stained for phospho-[Ser9]-GSK3β (P-GSK3β), with a specific rabbit primary antibody (Sigma-Aldrich), diluted at 1:200 and marked with Donkey anti-rabbit IgG conjugated with Alexa-488 (Thermo Fisher). Moreover, the colocalization of P-GSK3β with choline-acetyltransferase (ChAT), was also carried out in a different set of slices and only from N condition, using the already described primary Anti-P-GSK3β and secondary Alexa-488, together with a specific goat primary antibody for ChAT (Merk-Millipore), diluted at 1:300 and marked with Donkey anti-goat IgG conjugated with Alexa-555 (Thermo Fisher). The procedure steps were the same as those described earlier.

Microglia were specifically stained with the rabbit polyclonal Anti-Iba1 antibody (1:800; Wako) and the secondary antibody Anti-rabbit IgG conjugated with Alexa-488. The procedure was the same as that described earlier in the text.

### Image Acquisition and Analysis

Images were obtained with a Nikon eclipse 80i equipped with Nikon Digital Sight DS-Vi1 color camera, at 100x magnification (200x for the microglia staining). For each rat, among the different sections cut from the same SpCo tract and for both levels, those with the best qualities (i.e., homogeneous tissue, not cracked and with good staining) were chosen for the image acquisitions. This step consisted in taking three pictures of a single slice, both for cervicothoracic and for lumbar levels, as follows (see Figure 2): (i) one hemi-ventral part framed, including the ventral horn (VH); (ii) one hemi-central part framed, including the central canal (Central); (iii) one hemi-dorsal part framed, including the dorsal horn (DH). As shown in Figure 2, the frames of the pictures taken are partly overlapping, this choice was driven by: (i) the limitation given by the lowest microscope magnification useful to take a good fluorescent signal; (ii) the aim of analyzing the whole of the SpCo gray matter, since there are no previous data on this specific topic. The visual recognition of these structures was possible while observing the NeuN staining (Alexa-488) of the whole section and comparing it with the atlas schemes as well as with cresyl violet staining (Figure 2). It was possible to distinguish the different regions and levels of the SpCo by grossly evaluating, through observation, the white to gray matter ratio, which was clearly smaller in cervicothoracic sections (Paxinos and Watson, 2007), as shown in Figure 2.

Picture acquisition and analysis procedures have been already described (Luppi et al., 2019). Briefly, each microscopic field was acquired in both fluorochrome colors (i.e., Alexa-488 and Alexa-594 or Alexa-555) with two separate pictures (only one for microglia acquisition). The exposure time of the camera was manually regulated for each picture to the best of the experimenter's evaluations, with the aim of reproducing best that which could be observed directly through the oculars. The experimenter was blind to the experimental conditions. Considering the dark field of fluorescence images, the fine regulation of the exposure time for every picture was necessary to avoid automatic compensations of the camera that might have interfered with the subsequent evaluation of the staining intensity. All the other camera parameters were kept constant throughout the experiment. Thanks to the preview function of the camera, all this procedure took only a few seconds for each picture, avoiding any problem of fluorescence fading.

Two experimenters working in the same conditions independently carried out the evaluations of the staining intensity. PP-Tau accumulation was quantified in each AT8 and Tau-1 picture by the experimenter's subjective estimation of the staining intensity. The estimation was given while observing pictures on the monitor (using Windows Photo Viewer) and scoring the intensity within a range from “-” (completely absent) to “++++” (maximum staining), on a scale of 5 levels (cf. Luppi et al., 2019). The final score was obtained by averaging the scores given by the two experimenters and also averaging the two SpCo levels considered and all the animals belonging to the same experimental condition.

Staining for P-GSK3β/ChAT was qualitative and carried out on two samples from a single rat per experimental condition.

Microglia activation level was measured following established morphometric parameters (Davis et al., 2017; Baldy et al., 2018): (i) Soma area; (ii) Arborization area; (iii) Morphological index (MI): soma area/arborization area ratio; (iv) Microglial density (counting the number of cells in every picture taken); (v) Nearest neighbor distance. Measurements were carried out by means of Image Pro Analyzer 7.0 (Media Cybernetics) using the inbuilt calibration function.

### Statistical Analysis

#### PP-Tau Staining

The analysis consisted in two steps: (i) “gross analysis,” considering together the scores of all the structures analyzed; (ii) “fine analysis,” considering the three frames studied separately.

We used the non-parametric Kruskal-Wallis test and, only if the null hypothesis was rejected, pairwise comparisons were carried out using the non-parametric Mann-Whitney test for the following evaluations: (i) all the experimental conditions vs. C; (ii) R6 vs. ER; (iii) R6 vs. R38. Significance level was preset at *P* < 0.05 for all comparisons.

#### Microglia Analysis

This analysis was carried out with a one-way ANOVA, considering only C, N, R6, and R38 experimental conditions (see Figure 1), and independently for VH and DH of the SpCo (see Figure 2). In the case that the ANOVA was significant, the following *post-hoc* comparisons were carried out using the modified *t*-test (t^*^), with α level opportunely modified following the sequential Bonferroni method (Holm, 1979): (i) all the experimental conditions vs. C; (ii) R6 vs. N; (iii) R38 vs. N. Significance level was preset at *P* < 0.05 for all comparisons.

No statistical analysis was carried out for P-GSK3β/ChAT, which was intended as purely qualitative data.

## Results

**Figure 3:**
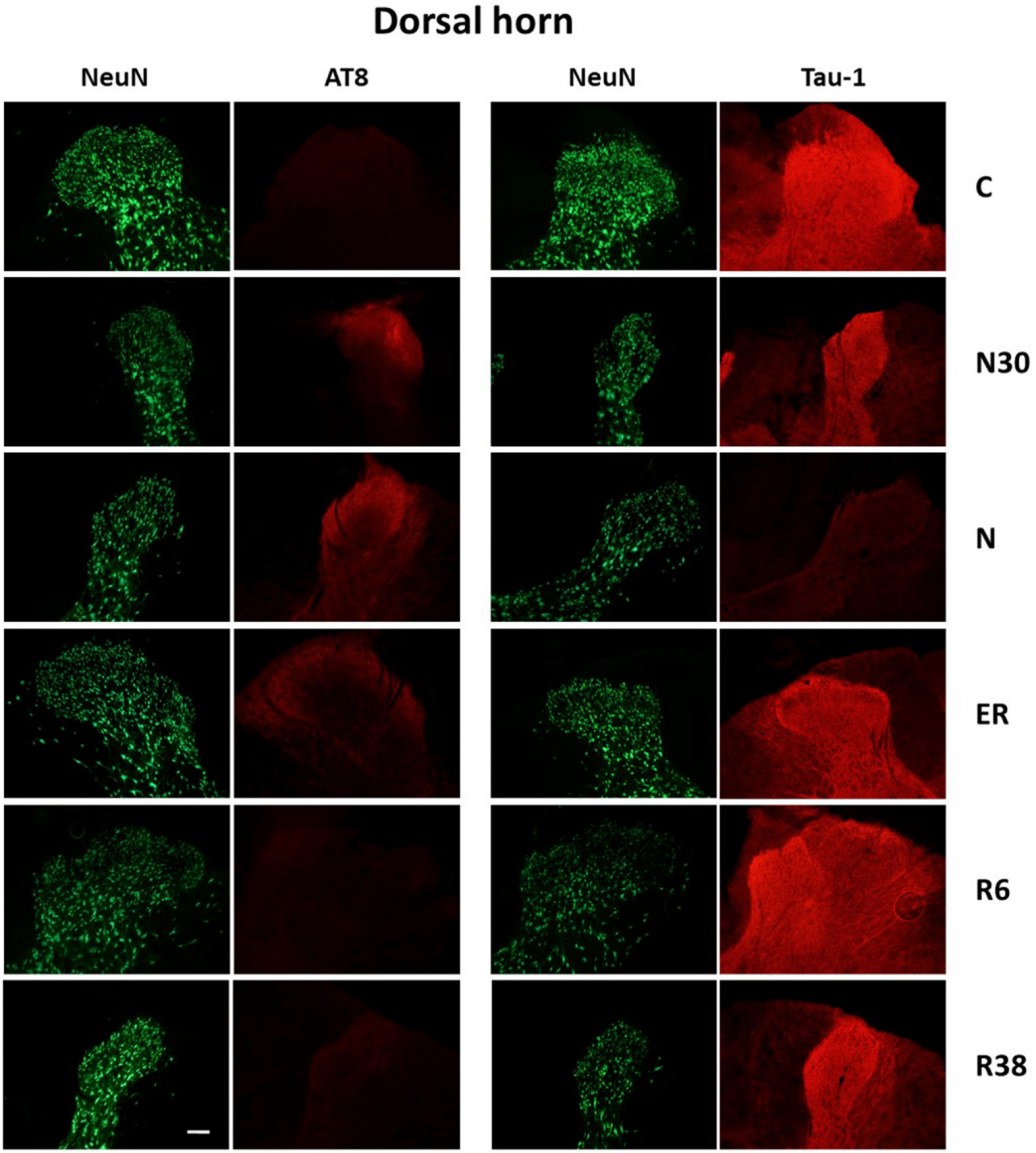
Representative pictures showing the dorsal horn of the spinal cord. Green pictures represent NeuN staining (neuronal maker, secondary conjugated with Alexa-488), for the recognition of the anatomical structure. In the left half of the figure, red pictures represent the same corresponding field depicted in the nearby NeuN column, but stained for AT8 (phosphorylated Tau, secondary conjugated with Alexa-594). In the right half of the figure, red pictures represent the same corresponding field depicted in the nearby NeuN column but stained for Tau-1 (non-phosphorylated Tau, secondary conjugated with Alexa-594). C, control; N30, samples taken during the induction of synthetic torpor (ST), when brain temperature (Tb) reached 30°C; N, samples taken at nadir of hypothermia, during ST; ER, early recovery, samples taken when Tb reached 35.5°C following ST; R6, samples taken 6 h after ER; R38, samples taken 38 h after ER. Calibration bar: 100 μm.

**Figure 4:**
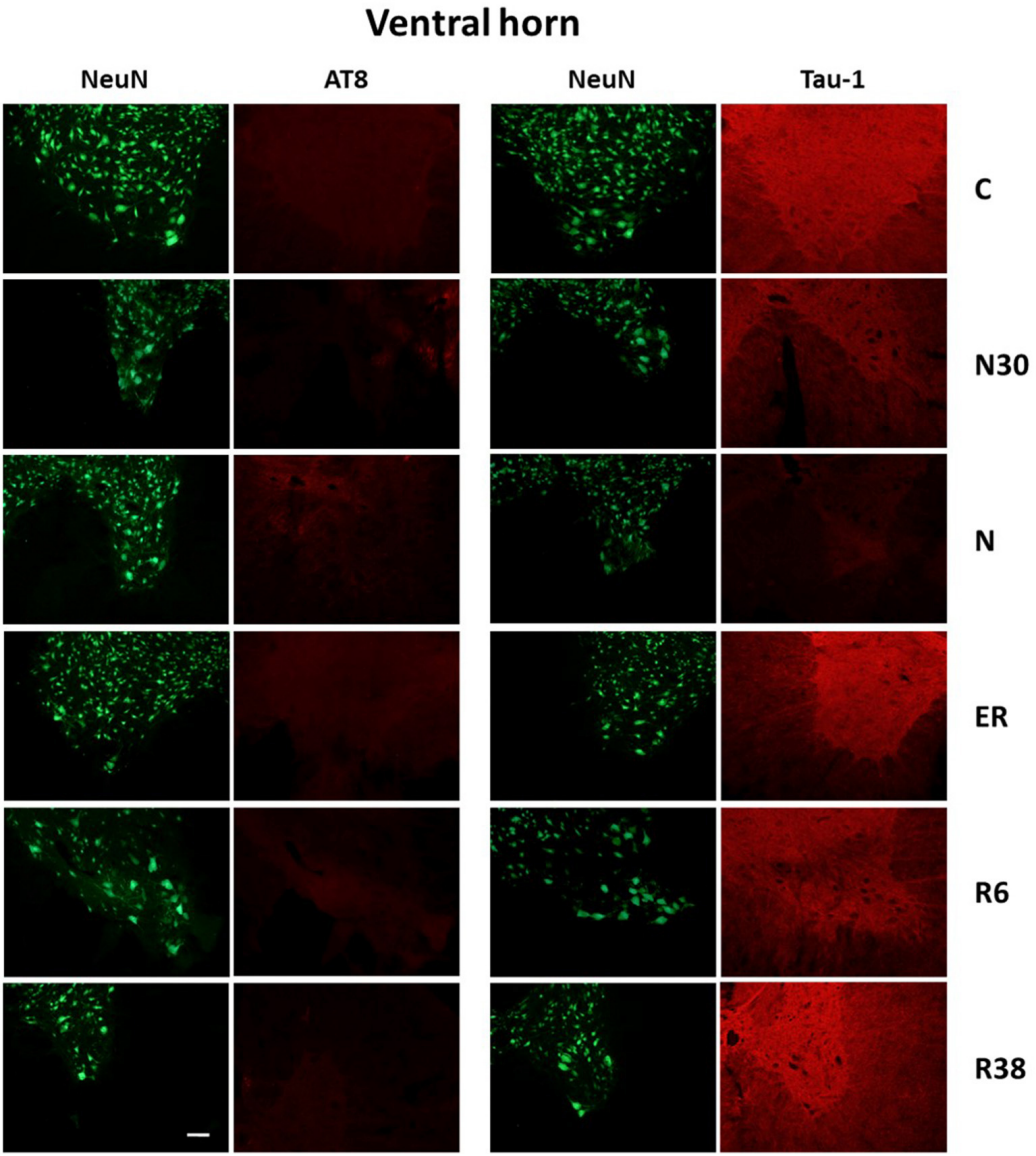
Representative pictures showing the ventral horn of the spinal cord. Green pictures represent NeuN staining (neuronal maker, secondary conjugated with Alexa-488), for the recognition of the anatomical structure. In the left half of the figure, red pictures represent the same corresponding field depicted in the nearby NeuN column, but stained for AT8 (phosphorylated Tau, secondary conjugated with Alexa-594). In the right half of the figure, red pictures represent the same corresponding field depicted in the nearby NeuN column but stained for Tau-1 (non-phosphorylated Tau, secondary conjugated with Alexa-594). C, control; N30, samples taken during the induction of synthetic torpor (ST), when brain temperature (Tb) reached 30°C; N, samples taken at nadir of hypothermia, during ST; ER, early recovery, samples taken when Tb reached 35.5°C following ST; R6, samples taken 6 h after ER; R38, samples taken 38 h after ER. Calibration bar: 100 μm.

Results are collected and shown in Table 1, while exemplificative image examples are presented in Figures 3, 4. All analyses were carried out by pooling the data from the cervicothoracic region with those from the lumbar level of the SpCo (Figure 2), since no differences were observed between the two levels for any of the parameters.

**Table 1:** AT8 (phosphorylated tau protein) and Tau-1 (non-phosphorylated tau protein) staining intensities.

**AT8**	**C**	**N30**	N^*^	**ER**	R6^*^^#^^§^	**R38**
VH	+	+	+	+	–^*^^#^^§^	+
Central	+	+	++^^*^^	+	–^*^^#^^§^	+
DH	+	++^^*^^	++^^*^^	+	–^*^^#^^§^	+
**Tau-1**	**C**	**N30**	N^*^	**ER**	**R6**	**R38**
VH	++	++	+^*^	++	+^*^^#^^§^	++
Central	++	+++	+^*^	+++	++	++
DH	+++	+++	+^*^	+++	+++	+++

*VH, ventral horn; Central, central part of the hemicoronal section of the spinal cord, in between the ventral and the dorsal horns; DH, dorsal horn (see Figure 2). Experimental conditions (column heads) are defined in the Methods section and in Figure 1*.

* vs. C;

# R6 vs. ER;

§ *R6 vs. R38*.

Considering the “*gross*” analysis, which was carried out in order to have a broad idea of the phosphorylation pattern of Tau in the SpCo taken as a whole, the immunoreactivity (ir) was significantly higher for AT8 (*P* < 0.001), and lower for Tau-1 (*P* < 0.001) in N compared to C. For AT8 alone, staining intensity was also significantly lower in R6, compared to C (*P* < 0.001), ER (*P* < 0.001), and R38 (*P* < 0.001).

The “*fine*” analysis, i.e., considering the dorsal horn (DH), central canal (Central), and ventral horn (VH) separately (see Figure 2), showed that AT8ir was significantly higher in N compared to C in Central (*P* = 0.004) and DH (*P* = 0.006), but, notably, no differences were found in VH (*P* = 0.418). In DH only, AT8ir was significantly higher (*P* = 0.014) in N30 than C. For the R6 condition, the differences in AT8ir were as follows: staining was significantly lower than C (VH: *P* = 0.006; Central: *P* = 0.002; DH: *P* < 0.001), ER (VH: *P* = 0.006; Central: *P* = 0.001; DH: *P* < 0.001), and R38 (VH: *P* < 0.001; Central: *P* < 0.001; DH: *P* = 0.002).

As far as Tau-1ir (Table 1) is concerned, a significantly lower intensity was observed in N vs. C for all the structures analyzed (VH: *P* = 0.002; Central: *P* < 0.001; DH: *P* < 0.001). Only in VH, was R6 significantly lower than C (*P* = 0.017), ER (*P* = 0.012), and R38 (*P* = 0.049).

**Figure 5:**
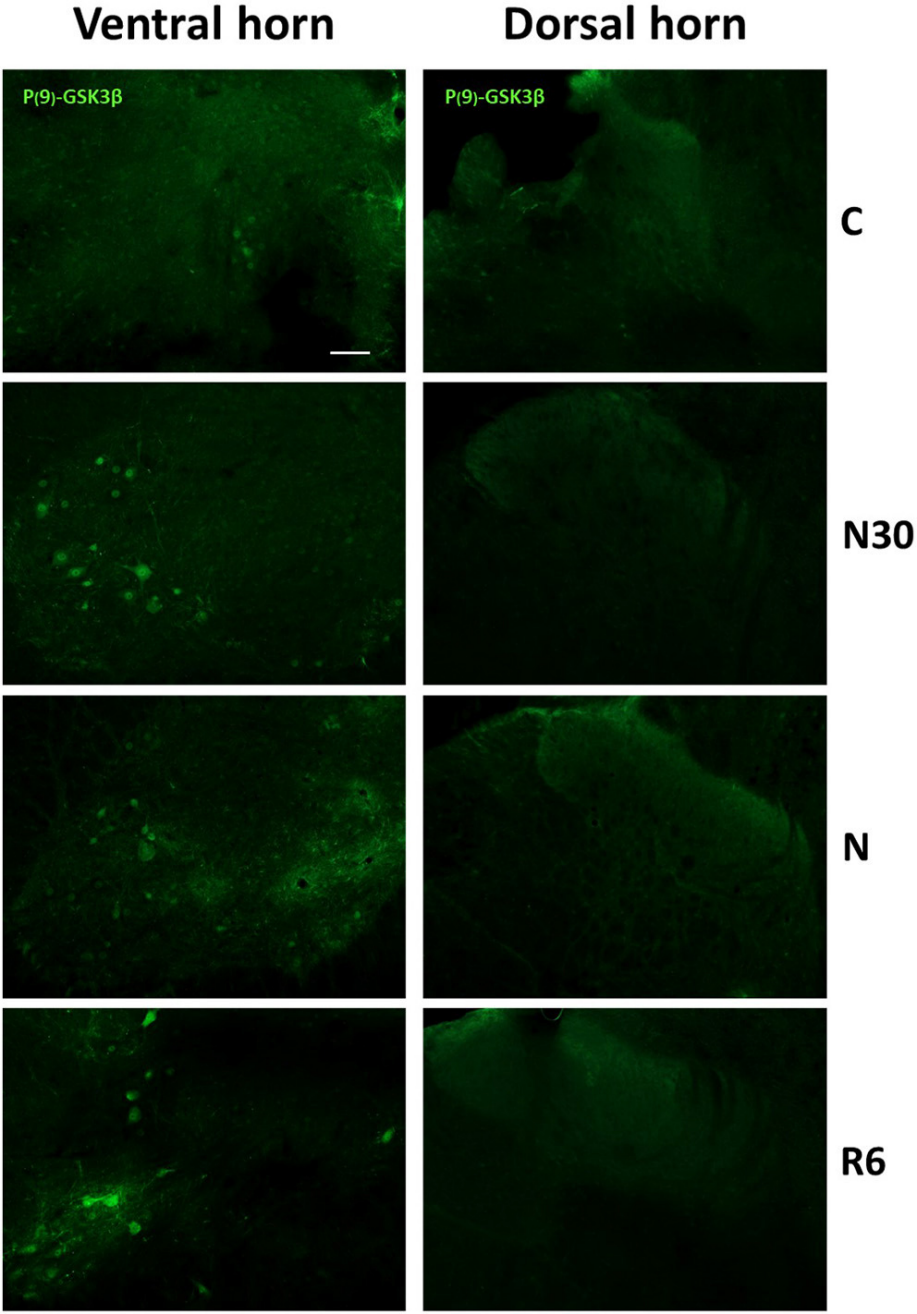
Representative pictures showing the staining for P(9)-GSK3β (inactive form, secondary conjugated with Alexa-488) are shown for the ventral (left) and dorsal (right) horns of the spinal cord. C, control; N30, samples taken during the induction of synthetic torpor (ST), when brain temperature (Tb) reached 30°C; N, samples taken at nadir of hypothermia, during the ST bout; R6, samples taken 6 h after Tb reached 35.5°C following ST. Calibration bar: 100 μm.

Since glycogen-synthase kinase-3-β (GSK3β) is recognized as the main kinase involved in PP-Tau accumulation (Planel et al., 2004), and the phosphorylation in Ser9 position (P-GSK3β) has inhibitory effects on this enzyme (Frame et al., 2001), to better explain results from AT8ir an immune-staining for P-GSK3β was also carried out. Results showed positive cells only within the VH (Figure 5), both during the induction of ST and during recovery, while no staining was found in the DH. These results are in line with the lack of AT8ir in VH.

**Figure 6:**
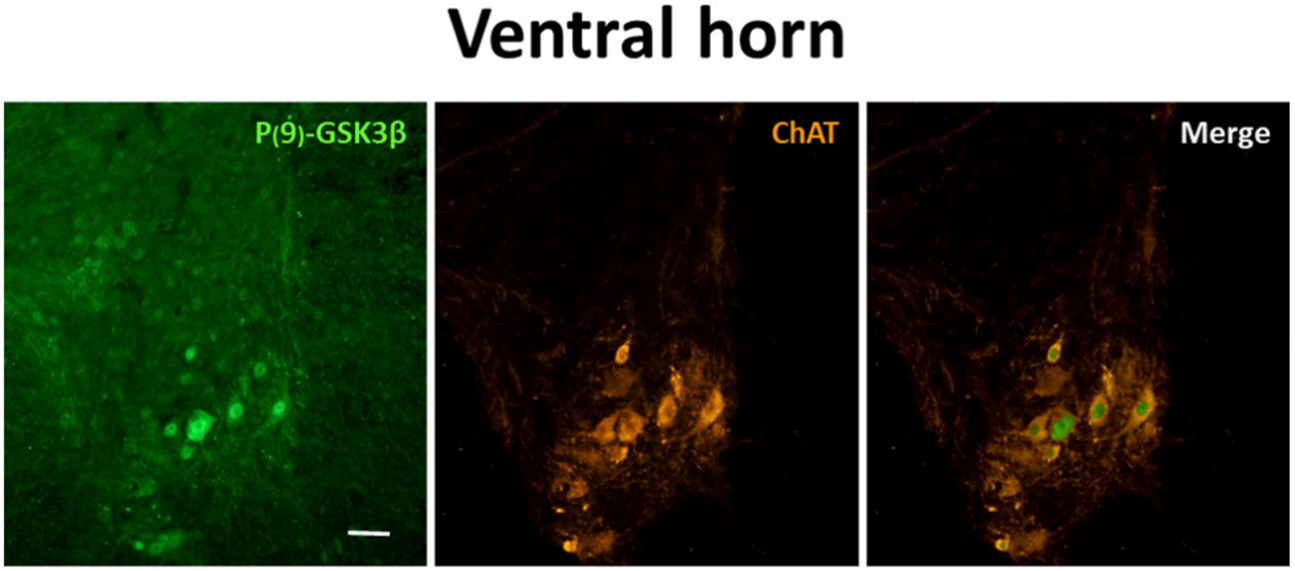
Representative pictures showing the double staining for P(9)-GSK3β (inactive form of GSK3β; left panel, secondary conjugated with Alexa-488), choline-acetyltransferase (ChAT; to mark motor neurons; central panel, secondary conjugated with Alexa-555) and merging (right panel). Pictures were taken during the synthetic torpor at nadir of hypothermia (N condition, see Figure 1), framing the only ventral horn of a cervicothoracic region of the spinal cord. Calibration bar: 100 μm.

Moreover, to verify whether the P-GSK3β-ir was specifically expressed in motor neurons, a double-staining experiment with ChAT was carried out only from N condition. Results showed a very frequent co-localization of the two antigens, as represented in Figure 6, indicating a specific inhibition of GSK3β in motor neurons of the SpCo during ST.

In Table 2 are shown results from the analysis of the microglia morphology, that was aimed at verifying the possible activation of a neuro-inflammatory response. Representative images are shown in Supplementary Figure 1. Microglia results showed very few significant differences throughout the experiment. In particular, only the MI was significantly lower (indicating a resting phenotype of the microglia) in N compared to C (*P* = 0.006) and R38 (*P* < 0.001): both differences were limited within only the DH [*F*_(3, 36)_ = 2.904; *P* = 0.048]. No statistically significant differences were found for any of the other morphometric parameters studied (Table 2).

**Table 2:** Microglia analysis.

	**C**		**N**		**R6**		**R38**	
	**VH**	**DH**	**VH**	**DH**	**VH**	**DH**	**VH**	**DH**
Cell count	76.1 ± 10.3	70.9 ± 7.2	60.5 ± 9.0	58.3 ± 5.3	63.3 ± 8.3	72.5 ± 3.5	59.8 ± 3.1	68.3 ± 7.1
SA (μm^2^)	46.7 ± 1.3	48.6 ± 2.7	43.5 ± 2.3	40.9 ± 2.6	46.7 ± 4.6	51.8 ± 3.2	46.6 ± 4.0	46.7 ± 3.0
AA (μm^2^)	826.1 ± 270.9	722.7 ± 210.6	608.2 ± 41.4	595.5 ± 29.0	534.8 ± 54.1	663.4 ± 70.7	501.2 ± 36.5	589.3 ± 101.5
MI	0.155 ± 0.047	0.150 ± 0.040	0.082 ± 0.004	0.076^*^±0.005	0.107 ± 0.014	0.096 ± 0.008	0.107 ± 0.011	0.100^§^±0.013
Nnd (μm)	41.1 ± 4.1	46.0 ± 4.0	43.9 ± 2.4	42.1 ± 2.0	46.0 ± 1.9	43.3 ± 2.2	40.8 ± 2.5	37.9 ± 3.0

*Morphometric parameters for the microglia analysis are shown as mean ± s.e.m. SA, soma area; AA, arborization area; MI, morphological index (SA/AA); Nnd, nearest neighbor distance; VH, ventral horn; DH, dorsal horn. Experimental conditions (in bold) are defined in the Methods section and in Figure 1*.

* vs. C;

§ *vs. N*.

## Discussion

The present results show that, similarly to what has been observed in the brain (Luppi et al., 2019), synthetic torpor induces a reversible accumulation of PP-Tau in the SpCo of rats, a non-hibernating species. However, different phosphorylation patterns were observed within the SpCo: surprisingly, no AT8ir was observed in the ventral horn (Table 1).

To the best of our knowledge, there are no data in literature describing the phosphorylation/dephosphorylation processes of Tau protein in the SpCo of mammals in natural or synthetic torpor; the present work, therefore, represents the first study on the topic. A relevant difference in PP-Tau pattern during ST was observed in DH compared to VH, while the pattern of the Central part appears to be intermediate, probably due to the fact that the slice-frame of the Central part largely overlaps with those of both the DH and the VH. In particular, while AT8ir was clearly increased by ST in the DH, this was not the case for VH. However, a significant decrease in Tau-1ir at the nadir of hypothermia was observed not only in the DH, but also in the VH. Thus, in the VH a reduction in the non-phosphorylated form of Tau occurred in the absence of a reciprocal increase of the Ser202/Thr205-phosphorylated form (Malia et al., 2016).

In our opinion, these results may have two important implications: (i) Tau protein is phosphorylated in different positions in VH and DH; (ii) neurons of the VH, also differently from those in many brain areas (Luppi et al., 2019), promote phosphorylation of Tau protein during ST in positions not recognized by the monoclonal AT8 antibodies, therefore showing some kind of “AT8 resistance.”

Although Tau protein may be phosphorylated in many amino-acidic sites (Wang and Mandelkow, 2016), AT8ir is widely used in studying tauopathies, as in Alzheimer's disease staging in humans (Braak et al., 2006). Dugger et al. (2013) showed AT8ir in SpCo of elderly people, being higher in Alzheimer's patients, but also suggesting that the accumulation of PP-Tau within the SpCo does not appear to be clinically relevant (Dugger et al., 2013). However, in Alzheimer's animal models PP-Tau in SpCo is well-described (Lewis et al., 2000; Allen et al., 2002). Our results show a lack of AT8ir in the VH during ST: this suggests that under ST Tau phosphorylation may take place in a different and peculiar way within the VH, at least considering the 6-h time window studied. As already discussed (Luppi et al., 2019), the possibility of a phosphorylation occurring only in the Threonine-205 position of Tau protein (which is not enough for AT8ir, but is enough to depress Tau-1ir) is intriguing, since this phosphorylated form of Tau resulted to be protective against neurodegeneration (Ittner et al., 2016), but other experiments are needed to verify this possibility.

We sought to delineate at least part of the mechanism underlining this peculiar data by staining SpCo slices for Phospho-Ser9-GSK3-β (P-GSK3β), i.e., the inactive form of the enzyme (Grimes and Jope, 2001). Glycose-syntase-kynase 3-β is considered the most important kinase phosphorylating Tau protein, although it is not the only one (Planel et al., 2001; Su et al., 2008; Wang and Mandelkow, 2016). In the present study, staining for P-GSK3β was positive during the hypothermic periods for the majority of the motor-neurons in the VH (Figures 5, 6), while no staining was observed within the DH (Figure 5). Thus, in the VH, a biochemically-regulated process of inhibition of GSK3β may lead to a “partial” phosphorylation of Tau protein, not involving all the positions necessary for recognition by the AT8 antibody (Malia et al., 2016).

Since the condition in question is one of suspended animation (Cerri et al., 2013), during ST motor activity is strongly lowered, while the peripheral sensory barrage should be active for a reasonably long time. An activity-dependent phosphorylation of GSK3β has been described in the suprachiasmatic nucleus (SCN) of mice, in a work showing that a light-pulse delivery during the dark period of the LD cycle (i.e., when SCN neurons are less active) induces the dephosphorylation of GSK3β (Paul et al., 2017). On these bases, we suggest that the strong reduction in the activity of VH motoneurons during ST may facilitate an active phosphorylation of GSK3β (i.e., with inhibiting effects of the kinase activity) in these cells, preserving them from the accumulation of AT8ir. Concomitantly, while body temperature drops, neurons in the DH should be more active than those in the VH, due to the cold-induced somatosensory inputs from periphery to laminae I-IV of the SpCo (Craig, 2002; Todd, 2017; Fernandes et al., 2018). With a sustained activity on neurons within the DH, the phosphorylation process of GSK3β may not take place with the same efficiency as with the poor activity that should characterize neurons within the VH. Similarly to the DH neurons, brain neurons should be more active than spinal motoneurons during ST. Even though it is not possible to prove the existence of such activity (in particular in diencephalic and brainstem structures) without direct recordings, it is worth noting that some EEG activity was observed during ST and was also found to be present when Tb reached the nadir of hypothermia (Cerri et al., 2013). This finding sustains the possibility of residual activity in the thalamo-cortical system under these extreme conditions. However, the eventual link between neuronal activity and the phosphorylation of both GSK3β and Tau protein within the SpCo should be the topic of future experiments.

The comparison of the present results with those observed in the brain of the same animals (Luppi et al., 2019) points to the existence of a different pattern of PP-Tau accumulation/resolution that is also present during the recovery period. In this condition, while in the brain the disappearance of AT8ir was not accompanied by the specular reappearance of Tau-1ir, in the SpCo the normalization of Tau-1ir was complete in all the areas analyzed, independently from the degree of AT8ir shown during ST. Thus, while in the brain the dephosphorylation of Tau may be interpreted as being targeted to AT8-specific epitopes of Tau monomers (possibly, to Ser202), this is apparently not the case for the SpCo, in which the dephosphorylation was completed within the residue window of 189–207 (i.e., for the Tau-1ir; Szendrei et al., 1993; Billingsley and Kincaid, 1997).

The reversibility of PP-Tau accumulation observed in the present work cannot exclude a certain rate of cell death following recovery from ST. However, since previously published behavioral data (Cerri et al., 2013) did not show significant gross neurological dysfunction, in our opinion neuronal apoptosis is unlikely to be stimulated. Anyway, direct measurements of pro- and anti-apoptotic factors are needed, and the topic undoubtedly is worth of dedicated future experiments.

We also investigated microglia activation within the SpCo, but our morphometric results show no meaningful changes. Small changes are shown in the MI parameter (Baldy et al., 2018), that evolves toward lower values and lesser variability during ST and the following recovery. A low MI means higher ramification, a sign of the resting phenotype of microglia cells (Graeber and Streit, 2010). This clearly shows that ST, while inducing dramatic changes in Tau phosphorylation, does not trigger any neuroinflammatory response within the SpCo, this being considered an important factor in neurodegenerative diseases (Ransohoff, 2016; Nilson et al., 2017). This is somehow different from what was found in the brain (Luppi et al., 2019), where apparently a transient gliosis was shown during the ST bout, rapidly returning to the normal condition during recovery from ST. It is worth noting that, as specified in the paper, those data were preliminary and still incomplete (Luppi et al., 2019).

In conclusion, the reversibility of PP-Tau during ST found in the SpCo, as well as in the brain (Luppi et al., 2019), suggests that, when specific conditions are met, even a non-hibernating mammal is able to cope with a huge PP-Tau accumulation, reversing toward a normal condition in a few hours. Notably, this must happen independently by the specific natural evolution of hibernation. In our opinion, this represents the possible existence of a physiological mechanism, never described before, that may contrast PP-Tau accumulation before the development of neurodegeneration. Hence, we consider that the present data enrich the knowledge of this physiological process. In fact, important differences emerged between the PP-Tau accumulation/resolution patterns observed within the SpCo and the brain. This discrepancy may be mostly explained by the existence of different regulatory mechanisms of Tau kinases (i.e., phosphorylation/dephosphorylation of GSK3β) and phosphatases in neurons belonging to different divisions of the central nervous system.

Understanding this newly-described physiological process may represent a possible new approach in helping to contrast tauopathies. This original approach might consist in finding a way to sustain this physiological mechanism, alongside the blocking of those pathological mechanisms that are triggered by PP-Tau itself and that lead to neurodegeneration.

## Data Availability Statement

The raw data supporting the conclusions of this article will be made available by the authors, without undue reservation.

## Ethics Statement

The animal study was reviewed and approved by National Health Authority and Central Veterinary Service of the University of Bologna.

## Author Contributions

ML, RA, and MC contributed conception and design of the study. TH, FS, AO, and EP performed the experiments and collected data. ML performed the statistical analysis. ML and RA wrote the first draft of the manuscript. All authors discussed the results, contributed to manuscript revision, and read and approved the submitted version.

## Conflict of Interest

The authors declare that the research was conducted in the absence of any commercial or financial relationships that could be construed as a potential conflict of interest.

## Abbreviations

aCSF: artificial cerebrospinal fluid
AD: Alzheimer's disease
C: control group of animal
Central: central canal of the spinal cord
ChAT: choline-acetyltransferase
DH: dorsal horns of the spinal cord
EEG: electroencephalogram
ER: early recovery
GSK3β: glycogen-synthase kinase-3-β
IF: immunofluorescence
ir: immunoreactivity
LD: light-dark
MI: morphological index
N: nadir of hypothermia, when synthetic torpor is complete
N30: deep brain temperature at 30°C, half way during the induction of synthetic torpor
PBS: sodium phosphate buffer in saline
P-GSK3β: glycogen-synthase kinase-3-β phosphorylated at Ser9
PP-Tau: hyperphosphorylated tau protein
R38: 38 h from early recovery
R6: 6 h from early recovery
SCN: suprachiasmatic nucleus
SpCo: spinal cord
ST: synthetic torpor
Ta: ambient temperature
Tau: tau protein
Tb: deep brain temperature
VH: ventral horns of the spinal cord.
