# Supplementary material for: Reversible Tau Phosphorylation Induced by Synthetic Torpor in the Spinal Cord of the Rat
Source: Front Neuroanat. 2021 Feb 2;15:592288. doi: 10.3389/fnana.2021.592288 (PMC7884466; doi:10.3389/fnana.2021.592288)

**Figure S1.** Representative pictures showing the staining for Iba1 (specific microglia marker, secondary conjugated with Alexa-488) in ventral (left panel) and dorsal (right panel) horns of the spinal cord. C, control; N, sample taken at nadir of hypothermia, during the ST; R6, sample taken 6h after 35.5 °C brain temperature (Tb) was reached; R38, sample taken 38h after reaching 35.5 °C Tb (see Figure 1). Calibration bar: 50µm.


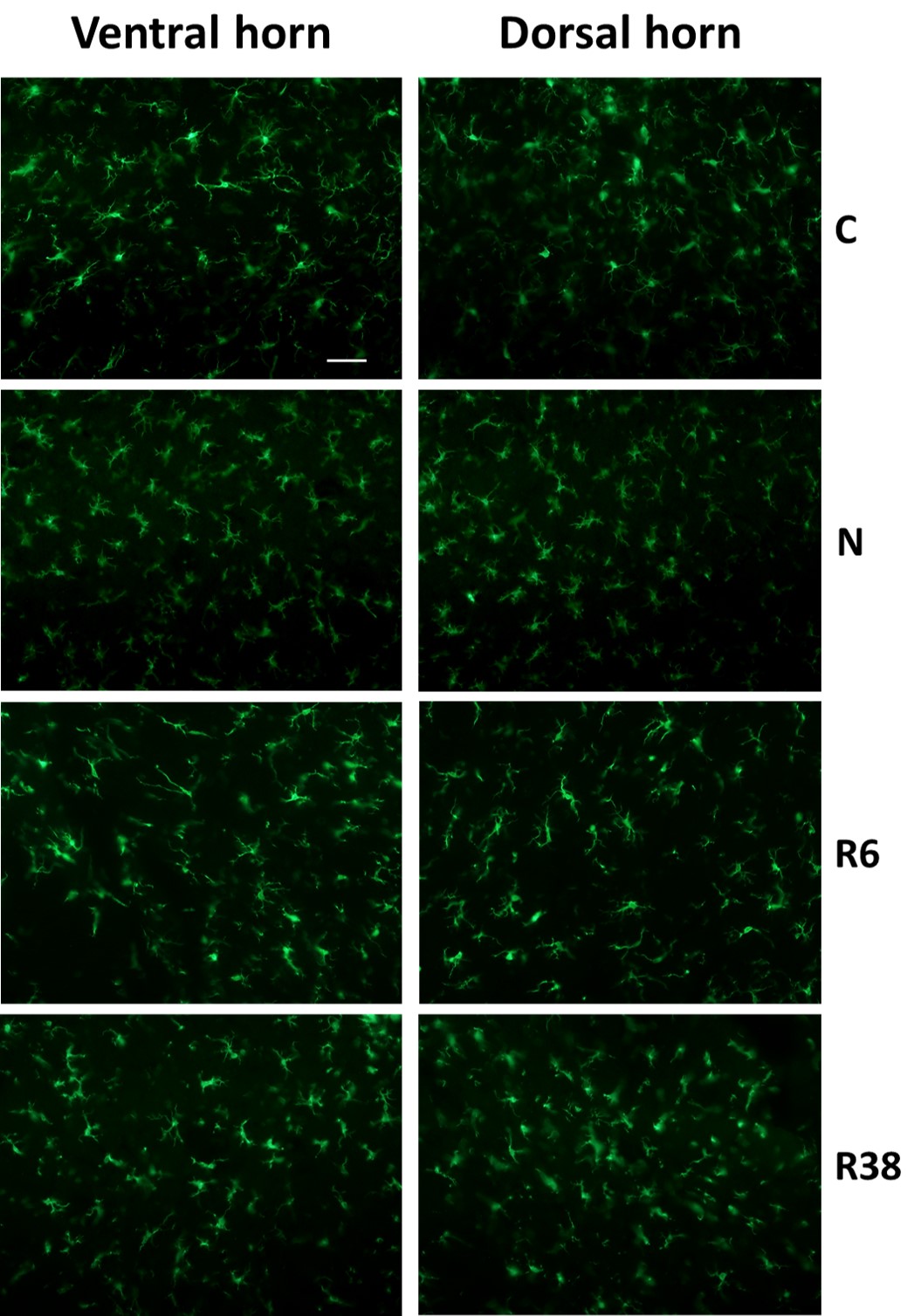

Supplement: Supplementary file 1 [file Table_1.DOCX]
